# Salivary cortisol differs with age and sex and shows inverse associations with WHR in Swedish women: a cross-sectional study

**DOI:** 10.1186/1472-6823-9-16

**Published:** 2009-06-21

**Authors:** Charlotte A Larsson, Bo Gullberg, Lennart Råstam, Ulf Lindblad

**Affiliations:** 1 University of Lund, Department of Clinical Sciences, Malmö, Community Medicine, Malmö, Sweden; 2 Department of Public Health and Community Medicine/Primary Health Care, The Sahlgrenska Academy at Gothenburg University, Gothenburg, Sweden; 3 Skaraborg Institute, Skövde, Sweden

## Abstract

**Background:**

Most studies on cortisol have focused on smaller, selected samples. We therefore aimed to sex-specifically study the diurnal cortisol pattern and explore its association with abdominal obesity in a large unselected population.

**Methods:**

In 2001–2004, 1811 men and women (30–75 years) were randomly selected from the Vara population, south-western Sweden (81% participation rate). Of these, 1671 subjects with full information on basal morning and evening salivary cortisol and anthropometric measurements were included in this cross-sectional study. Differences between groups were examined by general linear model and by logistic and linear regression analyses.

**Results:**

Morning and Δ-cortisol (morning – evening cortisol) were significantly higher in women than men. In both genders older age was significantly associated with higher levels of all cortisol measures, however, most consistently with evening cortisol. In women only, age-adjusted means of WHR were significantly lower in the highest compared to the lowest quartile of morning cortisol (p = 0.036) and Δ-cortisol (p < 0.001), respectively. Furthermore, when comparing WHR above and below the mean, the age-adjusted OR in women for the lowest quartile of cortisol compared to the highest was 1.5 (1.0–2.2, p = 0.058) for morning cortisol and 1.9 (1.3–2.8) for Δ-cortisol. All findings for Δ-cortisol remained after adjustments for multiple covariates and were also seen in a linear regression analysis (p = 0.003).

**Conclusion:**

In summary, our findings of generally higher cortisol levels in women than men of all ages are novel and the stronger results seen for Δ-cortisol as opposed to morning cortisol in the association with WHR emphasise the need of studying cortisol variation intra-individually. To our knowledge, the associations in this study have never before been investigated in such a large population sample of both men and women. Our results therefore offer important knowledge on the descriptive characteristics of cortisol in relation to age and gender, and on the impact that associations previously seen between cortisol and abdominal obesity in smaller, selected samples have on a population level.

## Background

Cortisol is a potent stress hormone and the secretion is regulated by the Hypothalamic-Pituitary-Adrenal-axis (HPA-axis). Cortisol is secreted in a specific diurnal pattern with a normal curve presenting a sharp peak in the early morning to then gradually decrease over the day and end up very low in the evening and at night. Except for the increased secretion in stressful situations, there are also smaller peaks during the day when the body is exposed to exercise, food, and tobacco [1]. Aging is hypothesised to alter the function of the HPA-axis in both men and women with increasing cortisol levels as a result, especially regarding nocturnal levels [2-7]. Previous studies have also indicated that cortisol levels differ between men and women [2-5,8,9]. However, while several of the studies have found men to have higher levels than women there are inconsistencies regarding in what age-groups these findings have been seen [2-5].

Patients with Cushing's syndrome are characterised by abdominal fat accumulation and decreased hip circumference, caused by their excess cortisol secretion. Consequently, numerous studies have investigated if increased cortisol levels are associated with abdominal obesity in otherwise healthy subjects too. While some studies have found total 24 h cortisol (urinary excretion or plasma/serum) to be positively correlated to abdominal obesity in women [10,11], others have found the opposite in women [12] or no association at all in either men [12,13] or women [14]. Furthermore, even though there are null findings too [10,15-18], morning cortisol seems to be negatively associated with abdominal obesity in both men and women [11,13,14,19-23].

The vast majority of previous studies of cortisol have focused on smaller selected groups. While these studies offer important contributions for understanding the underlying mechanisms of cortisol metabolism and its association with e.g. abdominal obesity, larger studies are needed to add information on what impact associations on smaller selected samples have on a population level. The present study was therefore designed to sex-specifically look at the diurnal salivary cortisol pattern under basal conditions and to explore the relationship between cortisol and abdominal obesity, in a large, randomly selected population.

## Methods

### Subjects

Vara is a small rural municipality in South-western Sweden with around 16 000 inhabitants. The population of Vara is a homogeneous population that generally resembles that of the total Swedish population. However, the Vara population has a lower level of education [24] and a lower level of foreign-born individuals [25]. Furthermore, both men and women in Vara are more obese [26], and women are less often smokers than the general Swedish population [24]. Between 2002 and 2004 a random sample from the Vara population was surveyed as part of a new population study within the Skaraborg Project. Participants were randomly selected, stratified by sex and five-year age groups, from all individuals between 30 and 74 years with a three times over-sampling in the ages 30 to 50 years as compared to those over 50 years. There were 1811 subjects who fulfilled all requirements for participation in the survey including visiting the study nurse, completion of the questionnaires, and having venous blood samples drawn (81% participation rate). After excluding a total of 140 subjects because of pregnancies (n = 5), use of cortisone medication (n = 10), missing waist circumference measurements (n = 1), and missing morning and/or evening salivary cortisol measurements (n = 124), 1671 subjects remained for further analyses in the present study. Informed consent was collected from all participants and the ethics committee at the University of Gothenburg, Sweden, approved the protocol.

### Procedures

Specially educated and trained nurses saw participants in the morning after an overnight fast (10 h). Participants signed an informed consent form and were then weighed on a calibrated scale and measured in light cloths and no shoes. They had their blood pressure taken twice in a supine position and had blood samples drawn. An oral glucose tolerance test (OGTT) was performed with an intake of 75 g standard glucose load [27]. In the two hours wait for the final blood drawing participants filled in a questionnaire regarding civil- and socioeconomic status, including educational level. Participants were also provided with a Salivette sampling device (cotton) along with both verbal and written instructions for usage. The instructions stated that participants were to: Collect saliva themselves at 0800 h and 2200 h (with a maximum of 30 minutes time shift) on one normal weekday within two weeks from the first study visit; abstain from food, drinks, snuff, smoking, tooth brushing, and exertion in the hour before saliva collection; rinse their mouths with water 15 minutes before the sampling; and rest for at least 15 minutes before sampling. Levels of saliva cortisol were analysed using a radioimmunoassay from Orion Diagnostica (Spectria™ Cortisol RIA) [28]. Approximately two weeks after the first visit the participants came for a second visit to the nurses to provide detailed information on medical history and ongoing medication, and to fill in a validated questionnaire regarding smoking habits, leisure time physical activity, and alcohol intake.

### Measures

Waist circumference was measured between the lowest rib margin and iliac crest and hip circumference at the largest circumference between waist and thighs. Waist-hip ratio (WHR) was defined as the ratio of waist to hip circumference.

Diurnal cortisol level (Δ-cortisol) was measured as the difference between logarithmic morning and evening saliva cortisol, which corresponds to the difference in percent between morning and evening values.

Current smoking was defined as daily smoking (yes/no). Leisure time physical activity was characterized based on four answer alternatives to the question "How physically active are you during your leisure time?". Alcohol consumption was defined by questions on how many days over the last 30 days that the subjects had consumed beer, wine, and strong liquor, respectively. Each of these questions was followed by questions on how many tins, glasses, and/or bottles that were normally consumed on such days. The total gram of alcohol consumed per week was then calculated by multiplying the number of days of alcohol drinking with the gram of alcohol that the items of consumed alcoholic beverage contain.

Educational level was examined by a question with 10 alternatives reaching from primary school to PhD-exams.

### Statistical analyses

SPSS Base System for Macintosh 11.0 was used for data analyses and all analyses were sex-specific. Baseline characteristics expressed as proportions were age-standardised in five-year intervals using the whole Vara population 30–75 years as standard. Differences between groups in continuous variables were examined by GLM (general linear model) and associations between continuous variables were analysed by linear regression. Associations between categorical variables were analysed by logistic regression and expressed as odds ratios (OR) with 95 per cent confidence intervals (CI). For the logistic regression analyses of the associations between WHR and morning cortisol/Δ-cortisol, WHR above and below the mean was used as dependent variable and quartiles of morning and Δ-cortisol, respectively, as independent variables. Pearson's correlation coefficient was used for testing the correlation between morning and evening cortisol. Confounding by differences in age, alcohol consumption, daily smoking (yes/no), leisure time physical activity, and use of oral contraceptives or estrogen replacements was controlled for in multivariate analyses and by stratification. Subjects treated with insulin were excluded from the analyses of 2 h blood glucose. Log transformation (10th logarithm) was used to induce normality in morning and evening cortisol. All tests were two-sided and statistical significance was assumed at p < 0.05.

## Results

The mean age in both men and women was 48 years (Table 1). Fasting blood glucose and blood pressure were higher in men and they reported lower levels of leisure time physical activity. Women were more often smokers than men and had higher salivary cortisol and 2 h glucose in an OGTT (Table 1).

**Table 1:** Characteristics in men and women

	**Women**			**Men**			
Characteristics	m.v.	m	sd (q1–q3)	m.v.	m	sd (q1–q3)	p-value
	**n = 838**			**n = 833**			
Age, years	0	48	12	0	48	12	0.875
Fasting p-glucose, mmol L^-1^	0	5.3	1.1	0	5.5	1.1	0.001
2 h p-glucose, mmol L^-1^	33	5.8	2.1	32	5.5	2.2	0.017
Systolic BP, mm Hg	0	121	14	0	125	14	<0.001
Diastolic BP, mm Hg	0	69	9	0	72	9	<0.001
Waist circumference, cm	0	85	12	0	95	12	<0.001
Hip circumference, cm	0	102	9	0	100	9	<0.001
WHR (waist-hip-ratio)	0	0.83	0.06	0	0.94	0.06	<0.001
BMI, kg m^-2^	1	26.8	4.5	0	26.9	4.6	0.442
Morning cortsiol, mmol L^-1^, all	0	12.5	(9.0–18.0)	0	11.1	(8.0–16.0)	<0.001
Morning cortsiol, <50 years	0	12.4	(9.0–18.0)	0	10.5	(7.0–15.0)	<0.001
Morning cortsiol, ≥ 50 years	0	12.5	(9.0–18.0)	0	12.1	(8.0–18.0)	0.491
Evening cortsiol, mmol L^-1^, all	0	2.3	(2.0–3.0)	0	2.2	(2.0–3.0)	0.132
Evening cortsiol, <50 years	0	2.2	(2.0–3.0)	0	2.1	(1.0–3.0)	0.386
Evening cortsiol, ≥ 50 years	0	2.7	(2.0–3.0)	0	2.5	(2.0–3.0)	0.183
Δ-cortisol, mmol L^-1^, all	0	5.3	(3.5–9.0)	0	5.0	(3.3–8.0)	0.086
Δ-cortisol, <50 years	0	5.8	(3.7–9.5)	0	5.1	(3.3–8.5)	0.004
Δ-cortisol, ≥ 50 years	0	4.6	(3.0–8.0)	0	4.8	(3.0–7.7)	0.469
Alcohol consumption, g/week^a^	6	22	(0–30)	2	61	(10–77)	<0.001^b^
	m.v.	n	%	m.v.	n	%	p-value
Oral contraceptives	-	64	6	-	-	-	-
HRT	-	26	5	-	-	-	-
Low physical activity, yes/no	8	50	7	11	67	7	0.094
Daily smoking, yes/no	3	182	21	4	128	15	0.001
Primary school only, yes/no	16	221	37	17	292	44	<0.001

All means (m) are adjusted for differences in age and proportions are standardised for age using the Vara population as standard. M.v. = missing values, P-glucose = plasma glucose, Δ-cortisol = logarithmic morning salivary cortisol – logarithmic evening saliva cortisol, HRT = hormone replacement therapy. For all cortisol variables means and quartiles 1–3 (q1–q3) are geometric (anti-log).

^a ^10 g alcohol is equivalent to approximately 1 glass of wine or 1 small beer.

^b ^The p-value is accounted for the generally higher physiological acceptance for alcohol in men as compared to women.

### Basal salivary cortisol

#### Age-differences

In both men and women evening cortisol was significantly higher in older subjects compared to younger, while the same pattern for morning cortisol was seen in men only (Table 2). In women Δ-cortisol was significantly lower in older subjects than in younger (Table 2). Test for trends over increasing age-groups, revealed highly significant trends (p ≤ 0.001) in all cortisol variables but morning cortisol in women (p = 0.215) and Δ-cortisol in men (p = 0.088).

**Table 2:** Male and female salivary cortisol levels in different age-groups.

	**Morning cortisol**				**Evening cortisol**				**Δ-cortisol**			
Age-group	n	m	q1–q3	p	n	m	q1–q3	p	n	m	q1–q3	p
**Women**												
30–39	242	12.4	9.0–17.0	ref.	242	1.7	1.0–3.0	ref.	242	5.8	3.7–10.0	ref.
40–49	291	12.4	8.0–19.0	0.892	291	2.2	2.0–3.0	0.834	291	5.7	3.7–9.0	0.768
50–59	146	11.6	8.7–15.2	0.260	146	2.5	2.0–3.0	0.030	146	4.7	3.0–8.0	0.005
60–69	107	12.7	10.0–18	0.732	107	2.7	2.0–4.0	0.004	107	4.7	3.3–7.5	0.015
≥ 70	52	15.1	9.2–19.8	0.029	52	3.6	2.0–6.0	<0.001	52	4.2	2.3–9.0	0.004
<50	533	12.4	9.0–18.0	ref.	533	2.2	1.4–3.0	ref.	533	5.8	3.7–9.5	ref.
≥ 50	305	12.5	9.0–18.0	0.773	305	2.7	2.0–3.0	<0.001	305	4.6	3.0–8.0	<0.001
**Men**												
30–39	254	10.4	7.0–15.0	ref.	254	2.0	1.0–3.0	ref.	254	5.3	3.5–9.0	ref.
40–49	276	10.6	8.0–15.0	0.634	276	2.2	2.0–3.0	0.101	276	4.9	3.0–8.0	0.288
50–59	141	11.8	8.0–18.0	0.030	141	2.4	2.0–3.0	0.007	141	5.0	3.0–8.0	0.460
60–69	106	12.5	8.0–17.3	0.006	106	2.5	2.0–3.0	0.001	106	4.9	3.3–7.6	0.459
≥ 70	56	12.3	9.0–16.8	0.049	56	2.9	2.0–4.0	<0.001	56	4.2	3.0–6.5	0.040
<50	530	10.5	7.0–15.0	ref.	530	2.1	1.0–3.0	ref.	530	5.1	3.3–8.5	ref.
≥ 50	303	12.1	8.0–18.0	0.001	303	2.5	2.0–3.0	<0.001	303	4.8	3.0–7.7	0.305

Δ-cortisol=logarithmic morning salivary cortisol-logarithmic evening salivary cortisol

Differences between age-groups were examined by GLM (general linear model), using group 30–39 and group <50, respectively, as reference. Means and quartiles 1–3 (q1–q3) are geometric (anti-log).

#### Sex-differences

Women in general were found to have significantly higher levels of morning cortisol than men, and women under <50 years were found to have higher levels of both morning and Δ-cortisol than corresponding men (Table 1). The correlation (r) between morning and evening cortisol values were 0.297 (p < 0.001) for women and 0.240 (p < 0.001) for men.

### Associations between salivary cortisol and abdominal obesity

**Figure 1:**
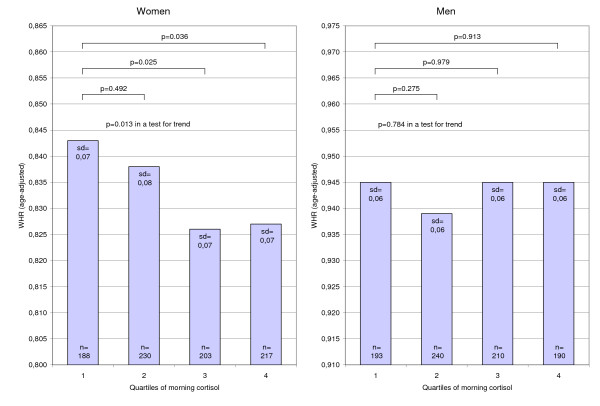
**Age-adjusted means of WHR (waist-hip ratio) by quartiles of morning cortisol**.

**Figure 2:**
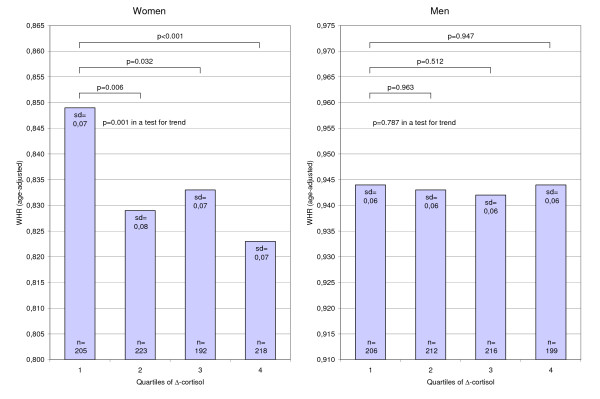
**Age-adjusted means of WHR (waist-hip ratio) by quartiles of Δ-cortisol (logarithmic morning cortisol – logarithmic evening cortisol)**.

In women, the age-adjusted mean of WHR was significantly lower in the two highest quartiles of morning cortisol compared to the lowest quartile (Figure 1 and Table 3). This association remained when also adjusting for leisure time physical activity, smoking, education, alcohol consumption, oral contraceptives, and estrogen replacements (Table 3), and was also seen in both a linear (p = 0.006) and a logistic regression analysis (OR 1.5, 1.0–2.2, p = 0.058). Similarly, the mean of WHR in the lowest quartile of Δ-cortisol in women was significantly higher than in the other three quartiles in the age-adjusted model (Figure 2 and Table 4), however, in the multivariate model the increase was only statistically significant in comparison with the second and the forth quartile (Table 4). The association between Δ-cortisol and WHR was also seen in women both above and below 50 years of age (data not shown). Moreover, a significant association between Δ-cortisol and WHR was seen in a linear regression analysis (p = 0.003) and in a logistic regression analysis (OR 1.9, 1.3–2.8), which both remained statistically significant when adjusting for multiple variables (Table 4). BMI did not affect any of the results above (data not shown).

**Table 3:** Comparisons of body composition between quartiles of morning salivary cortisol in men and women.

	**WHR**				**Waist circumference**			
	n	m	sd	p	n	m	sd	p
**Women**								
Adjusted for age								
qrtl 1 morning cortisol	188	0.843	0.069	ref.	188	86.63	13.52	ref.
qrtl 2 morning cortisol	230	0.838	0.076	0.492	230	85.90	13.53	0.585
qrtl 3 morning cortisol	203	0.826	0.071	0.025	203	83.99	13.52	0.055
qrtl 4 morning cortisol	217	0.827	0.074	0.036	217	84.74	13.54	0.163
**Test for linearity:**								
between quartiles				0.013				0.079
continuously				0.006				0.024
Adjusted for model 2^a^								
qrtl 1 morning cortisol	176	0.842	0.080	ref.	188	86.11	13.82	ref.
qrtl 2 morning cortisol	224	0.836	0.075	0.379	230	85.66	13.53	0.740
qrtl 3 morning cortisol	198	0.826	0.070	0.038	203	84.04	13.55	0.137
qrtl 4 morning cortisol	210	0.827	0.072	0.038	217	84.49	13.60	0.237
**Test for linearity:**								
between quartiles				0.019				0.135
continuously				0.012				0.075
**Men**								
Adjusted for age								
qrtl 1 morning cortisol	193	0.945	0.056	ref.	193	94.61	10.03	ref.
qrtl 2 morning cortisol	240	0.939	0.062	0.275	240	93.96	9.99	0.498
qrtl 3 morning cortisol	210	0.945	0.058	0.979	210	94.55	10.00	0.950
qrtl 4 morning cortisol	190	0.945	0.055	0.913	190	94.92	9.99	0.763
**Test for linearity:**								
between quartiles				0.784				0.613
continuously				0.980				0.556

Differences between quartiles (qrtl) of morning cortisol were examined by GLM (general linear model), using quartile 1 as reference.

^a^Model 2 = age, leisure time physical activity, smoking, education, alcohol consumption, oral contraceptives, and estrogen replacements.

**Table 4:** Comparisons of body composition between quartiles of Δ-cortisol in men and women.

	**WHR**				**Waist circumference**			
	n	m	sd	p	n	m	sd	p
**Women**								
Adjusted for age								
qrtl 1 Δ-cortisol	205	0.849	0.072	ref.	205	87.56	13.50	ref.
qrtl 2 Δ-cortisol	223	0.829	0.075	0.006	223	84.26	13.47	0.012
qrtl 3 Δ-cortisol	192	0.833	0.069	0.032	192	86.04	13.45	0.262
qrtl 4 Δ-cortisol	218	0.823	0.074	<0.001	218	83.59	13.47	0.003
**Test for linearity:**								
between quartiles				0.001				0.016
continuously				0.003				0.041
Adjusted for model 2^a^								
qrtl 1 Δ-cortisol	194	0.846	0.070	ref.	194	86.70	13.34	ref.
qrtl 2 Δ-cortisol	217	0.829	0.074	0.018	217	84.19	13.29	0.056
qrtl 3 Δ-cortisol	181	0.833	0.081	0.104	181	86.13	13.33	0.680
qrtl 4 Δ-cortisol	216	0.824	0.073	0.003	216	83.56	13.30	0.018
**Test for linearity:**								
between quartiles				0.009				0.071
continuously				0.009				0.119
**Men**								
Adjusted for age								
qrtl 1 Δ-cortisol	206	0.942	0.057	ref.	206	94.11	9.99	ref.
qrtl 2 Δ-cortisol	212	0.942	0.058	0.963	212	94.22	9.99	0.912
qrtl 3 Δ-cortisol	216	0.946	0.059	0.512	216	94.50	9.98	0.684
qrtl 4 Δ-cortisol	199	0.942	0.056	0.947	199	95.12	10.02	0.307
**Test for linearity:**								
between quartiles				0.787				0.291
continuously				0.957				0.310

Differences between quartiles (qrtl) of Δ-cortisol (logarithmic morning salivary cortisol – logarithmic evening salivary cortisol) were examined by GLM (general linear model), using quartile 1 as reference.

^a^Model 2 = age, leisure time physical activity, smoking, education, alkohol consumption, oral contraceptives, and estrogen replacements.

When WHR was substituted by waist circumference in each of the analyses above, the patterns generally remained, however, with less degree of statistical significance (Table 3 and 4). In men, morning cortisol and Δ-cortisol were not associated with either WHR or waist circumference (Figure 1 and 2, Table 3 and 4).

**Figure 3:**
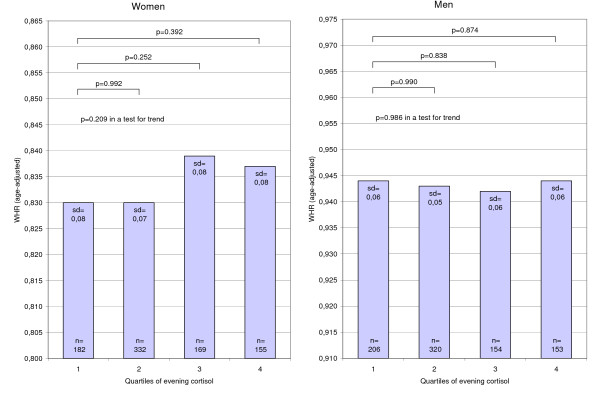
**Age-adjusted means of WHR (waist-hip ratio) by quartiles of evening cortisol**.

For evening cortisol there was no significant association with WHR (Figure 3 and Table 5) or waist circumference in either women or men (Table 5). However, in women both morning (p = 0.001) and evening values (p = 0.049) were significantly associated with WHR when simultaneously entered in an age-adjusted linear regression model. This association was not seen for waist circumference.

**Table 5:** Comparisons of body composition between quartiles of evening cortisol in men and women.

	**WHR**				**Waist circumference**			
	n	m	sd	p	n	m	sd	p
**Women**								
Adjusted for age								
qrtl 1 evening cortisol	182	0.830	0.081	ref.	182	85.03	13.60	ref.
qrtl 2 evening cortisol	332	0.830	0.073	0.992	332	84.80	13.54	0.851
qrtl 3 evening cortisol	169	0.839	0.078	0.252	169	86.13	13.56	0.451
qrtl 4 evening cortisol	155	0.837	0.075	0.392	155	85.78	13.61	0.617
**Test for linearity:**								
between quartiles				0.209				0.402
continuously				0.288				0.695
**Men**								
Adjusted for age								
qrtl 1 evening cortisol	206	0.944	0.057	ref.	206	95.43	10.08	ref.
qrtl 2 evening cortisol	320	0.943	0.054	0.874	320	94.19	9.96	0.167
qrtl 3 evening cortisol	154	0.942	0.062	0.838	154	93.63	10.00	0.095
qrtl 4 evening cortisol	153	0.944	0.062	0.990	153	94.66	10.03	0.478
**Test for linearity:**								
between quartiles				0.986				0.382
continuously				0.933				0.500

Differences between quartiles (qrtl) of evening cortisol were examined by GLM (general linear model), using quartile 1 as reference.

## Discussion

In this cross-sectional study we found significant age- and sex-differences in diurnal cortisol levels. Levels were generally higher in women than men and in older subjects compared to younger. We also found abdominal obesity to be significantly associated with low morning cortisol and low diurnal variation of cortisol, but only in women. To our knowledge, these associations have never before been investigated in such a large population sample of both men and women.

### Basal salivary cortisol

#### Age-differences

In the present study age was associated with higher morning cortisol in men and with higher evening cortisol in both men and women. Furthermore, age and Δ-cortisol were significantly inversely associated in women. This age-related increase in cortisol levels is supported by findings from several other studies, especially regarding nocturnal levels [2-7]. It has been concluded that while the basal secretion of cortisol remains fairly stable with age the negative feedback regulation of the HPA-axis seems to become impaired in older subjects [29,30]. This would lead to prolonged periods of increased cortisol secretion in response to for example stress and, thus, also increased general cortisol levels.

#### Sex-differences

We also found a consistent pattern of higher cortisol levels in women compared to men. While this have been seen in a couple of previous studies on older subjects [8,9], it is in contrast with others where men generally have been found to have higher cortisol levels [2,3,5]. However, in the latter studies there are some inconsistencies regarding in what age-groups the sex-differences have been seen. Different measurements of cortisol might be a plausible explanation for the incongruity between studies as some studies have measured the total cortisol levels in plasma or serum [2,5] while we and others [3] have measured the free cortisol levels found in saliva. This notion is supported by the findings of Kudelka et al [4] showing that while the total plasma cortisol seemed higher in older women than older men there was a significant reverse association regarding salivary cortisol. However, the analyses performed with saliva cortisol in previous studies [3,4] are still in contrast with our own results and the analyses with plasma cortisol [2,4,5] have not produced consistent results either, indicating other mechanisms behind the differing results. One might be that measurements of cortisol have been performed at different times of the day in different studies. Van Cauter et al [2] have found that the quiescent period tends to start earlier in women than in men. In two of the other previous studies [3,4] cortisol was in fact measured during the late afternoon when levels might have started sinking in women but not in men, thus, possibly explaining the higher levels found in men than women in these studies. Lastly, differences might partly be explained by most previous studies having used small, selected study groups, which make the results more unreliable and less representative of the general population.

### Associations between salivary cortisol and abdominal obesity

#### Women

The significant inverse association seen in women between morning cortisol and WHR is in concordance with other studies [11,14,19,20], of which two have used salivary cortisol like us [14,19]. Thus, the fact that saliva cortisol, in contrast to plasma or serum cortisol, primarily reflects free and active cortisol does not seem to affect the association seen between abdominal obesity and cortisol. This inverse association may instead be explained by an enhanced local clearance rate of cortisol in visceral fat depots since this type of fat has been found to harbour more glucocorticoid receptors than the subcutaneous type [31]. Higher metabolic transition rate of cortisol to its inactive metabolites [32] and impaired regeneration of cortisol from cortisone [33,34], have also been found in obese women compared to lean women. The enhanced clearance rate of cortisol is hypothesized to impair the negative feedback control of the HPA-axis and lead to an increased cortisol secretion [32,35]. This theory is supported by findings of increased total 24 h urinary cortisol out-put concurrent with decreased morning serum cortisol levels in abdominal obese subjects [11], and would explain our findings of abdominal fat accumulation in spite of low morning levels. Furthermore, higher cortisol response to both physical and mental stress has been found in women with high WHR compared to those with normal WHR [11,36]. Taken together, the findings indicate an abnormal activation of the HPA-axis in abdominally obese women. However, since there are also contradicting findings, the need for further studies, especially larger prospective ones, is vital. An alternative hypothesis may be that some of our abdominally obese women with low morning cortisol have been exposed previously to excessive cortisol output in response to stress, which caused their increased WHR. If the stress they experienced became chronic it might eventually have lead to the low cortisol levels seen, since chronic stress have been found to lead to an abnormal habituation to stress and ultimately hypocortisolism through dysregulation on some level of the HPA-axis [37,38].

In contrast to the morning values there was no significant association between WHR and evening cortisol in women. This is, however, not surprising since there is a lower variability in the evening values than in the morning values. Consequently, the inverse association between WHR and Δ-cortisol found in women is mainly an effect of the morning values. However, as the combined regression analysis of morning and evening cortisol with WHR revealed, evening values are also of importance for the association with abdominal obesity in women. The stronger association found between WHR and Δ-cortisol also emphasizes that an intra-individual statistical analysis of cortisol levels for detecting associations with abdominal obesity is preferred to an inter-individual analysis where morning and/or evening cortisol values are used separately.

Similarly to the association between WHR and cortisol, a significant inverse association was seen between waist circumference and Δ-cortisol in women, as well as a consistent tendency of an association between waist circumference and morning cortisol. However, WHR was clearly a stronger variable than waist circumference in the association with cortisol. Interestingly, this is in agreement with the body constitution of patients with Cuching's syndrome, who not only accumulate fat around the stomach but often also have decreased hip circumference.

### Men

The association between WHR and morning cortisol/Δ-cortisol found here in women was not duplicated in men and differences in the metabolism of cortisol could possibly hold some of the explanation. For example, while the metabolic clearance rate of cortisol has been found to be significantly higher in both obese men and women compared to lean subjects [29,33], some studies have only seen this in women [12,34]. Still, there are studies that also in men have found inverse associations between morning cortisol and WHR [13,14,21,22], as well as between morning cortisol and BMI [23]. Furthermore, while the reasons for the null-findings in men in previous studies are not known, it might be explained in the present study by our men's lower prevalence and variation of abdominal obesity compared to our women and consequently a decreased ability to identify an association with morning and Δ-cortisol.

### Methodological considerations

The main advantages of the present study are the large unselected study sample and the high participation rate (81%), which supports the representativeness of the study sample. There are nevertheless some potential limitations to the study: Firstly, the participants collected the saliva samples themselves and it is not unlikely that some participants have failed to fully comply with the instructions. Secondly, the morning cortisol values are based on a single measurement in our study. Since cortisol levels rise very sharply in the morning there is a risk of misclassification due to lack of adjustment for time of awakening and actual time of sampling. However, there is no reason to believe that these potential sources of misclassification would be systematic, and if anything it would only have weakened our findings. Thirdly, we don't know to what extent waist circumference and WHR depend on visceral fat tissue as opposed to the subcutaneous type. However, while waist circumference has mostly been found to be a better estimate of visceral obesity than WHR [39], the correlation between visceral obesity and WHR has still been good [40] and we therefore feel fairly confident that this is the case in our population too. Furthermore, we did look at cortisol levels in relation to both waist circumference and WHR in our study and WHR came out strongest. Fourthly, we were not able to control for menstrual cycle in women or reproductive hormones in general. Again, this limitation is more likely to have lead to type 2 errors than type 1 errors. In addition, our findings are supported by the presence of an association between WHR and Δ-cortisol in women over as well as under 50 years of age. The findings also remained in stratified analyses of estrogen consumption (data not shown). Lastly, the cross-sectional design makes it impossible to establish causality. In this case we cannot be sure whether decreased cortisol levels lead to abdominal fat accumulation or vice versa, which could have been decided on in a prospective study. Still, these results lay a solid foundation for hypotheses to be tested in future studies.

## Conclusion

While our findings of an age-related increase in basal cortisol levels are in concordance with previous studies, the results of generally higher cortisol levels in women than men of all ages are novel. We also confirm earlier findings of an inverse association between morning cortisol and abdominal obesity, however, only in women. Future studies should focus on intra-individual analysis and probably also account for reproductive hormones and stress exposure, as well as 24-h urinary cortisol and metabolites. Moreover, the possible association between cortisol and hip circumference needs to be further explored. To our knowledge, the associations in this study have never before been investigated in such a large population sample of both men and women. Our results therefore offer important knowledge on the descriptive characteristics of cortisol in relation to age and gender, and on the impact that associations previously seen between cortisol and abdominal obesity in smaller, selected samples have on a population level.

## Competing interests

The authors declare that they have no competing interests.

## Authors' contributions

CAL prepared the data, performed the statistical analyses, drafted the manuscript, and took part in conceiving the study. BG offered statistical expertise and performed some of the statistical analyses. LR conceived the study and acquired the data. UL conceived and coordinated the study, and acquired the data. All authors took part in the design of the study, the interpretation of data, the revision of the manuscript, and read and approved the final manuscript.

## Pre-publication history

The pre-publication history for this paper can be accessed here:



## References

[B1] Gallagher TF, Yoshida K, Roffwarg HD, Fukushima DK, Wetzman ED, Hellman L (1973). ACTH and cortisol secretory patterns in man. J Clin Endocrinol Metab.

[B2] Van Cauter E, Leproult R, Kupfer DJ (1996). Effects of gender and age on the levels and circadian rhytmicity of plasma cortisol. J Clin Endocrinol Metab.

[B3] Seeman TE, Singer B, Wilkinson CW, McEwen B (2001). Gender differences in age-related changes in HPA axis reactivity. Psychoneuroendocrinology.

[B4] Kudielka BM, Buske-Kirschbaum A, Hellhammer DH, Kirschbaum C (2004). HPA axis responses to laboratory psychosocial stress in healthy elderly adults, younger adults, and children: impact of age and gender. Psychoneroendocrinology.

[B5] Zhao Z-Y, Lu F-H, Xie Y, Fu Y-R, Bogdan A, Touitou Y (2003). Cortisol secretion in the elderly. Influence of age, sex and cardiovascular disease in a Chinese population. Steroids.

[B6] Giordano R, Bo M, Pellegrino M, Vezzari M, Baldi M, Picu A, Balbo M, Bonelli L, Migliaretti G, Ghigo E, Arvat E (2005). Hypothalamus-pituitary-adrenal hyperactivity in human aging is partially refractory to stimulation by mineralocorticoid receptor blockade. J Clin Endocrinol Metab.

[B7] Ferrari E, Cravello L, Muzzoni B, Casarotti D, Paltro M, Solerte SB, Fioravanti M, Cuzzoni G, Pontiggia B, Magri F (2001). Age-related changes of the hypothalamic-pituitary-adrenak axis: pathophysiological correlates. Eur J Endocrinol.

[B8] Laughlin GA, Barrett-Connor (2000). Sexual dimorphism in the influence of advanced aging on adrenal hormone levels: The Rancho Bernardo Study. J Clin Endocrinol Metab.

[B9] Gusenoff JA, Harman M, Veldhuis JD, Jayme JJ, St Clair C, Münzer T, Christmas C, O'Connor KG, Stevens TE, Bellantoni MF, Pabst K, Blackman MR (2001). Cortisol and GH secretory dynamics, and their interrelationships, in healthy aged women and men. Am J Physiol Endocrinol Metab.

[B10] Pasquali R, Cantobelli S, Casimirri F, Capelli M, Bortoluzzi L, Flamia R, Labate AM, Barbara L (1993). The hypothalamic-pituitary-adrenal axis in obese women with different patterns of fat distribution. J Clin Endocrinol Metab.

[B11] Mårin P, Darin N, Amemiya T, Andersson B, Jern S, Björntorp P (1992). Cortisol secretion in relation to body fat distribution in obese premenopausal women. Metabolism.

[B12] Strain GW, Zumoff B, Kream J, Strain JJ, Levin J, Fukushima D (1982). Sex differences in the influence of obesity on the 24 hr mean plasma concentration of cortisol. Metabolism.

[B13] Ljung T, Holm G, Friberg P, Andersson B, Bengtsson BA, Svensson J, Dallman M, McEwen B, Björntorp P (2000). The activity of the hypothalamic-pituitary-adrenal axis and the sympathetic nervous system in relation to waist/hip circumference ratio in men. Obes Res.

[B14] Duclos M, Pereira PM, Barat P, Gatta B, Roger P (2005). Increased cortisol bioavailability, abdominal obesity, and the metabolic syndrome in obese women. Obes Res.

[B15] Pasquali R, Anconetani B, Chattat R, Biscotti M, Spinucci G, Casimirri F, Vicennati V, Carcello A, Labate AM (1996). Hypothalamic-pituitary-adrenal axis activity and its relationship to the autonomic nervous system in women with visceral and subcutaneous obesity: effects of the corticotropin-releasing factor/arginine-vasopressin test and of stress. Metabolism.

[B16] Kajantie E, Eriksson J, Osmond C, Wood PJ, Forsén T, Barker DJ, Phillips DI (2004). Size at birth, the metabolic syndrome and 24-h salivary cortisol profile. Clin Endocrinol.

[B17] Duclos M, Gatta B, Corcuff J-B, Rashedi M, Pehourcq F, Roger P (2001). Fat distribution in obese women is associated with subtle alterations of the hypothalamic-pituitary-adrenal axis activity and sensitivity to glucocorticoids. Clin Endocrinol.

[B18] Kidambi S, Kotchen JM, Grim CE, Raff H, Mao J, Singh RJ, Kotchen TA (2007). Associations of adrenal steroids with hypertension and the metabolic syndrome in blacks. Hypertension.

[B19] Duclos M, Cordcuff J-B, Etcheverry N, Rashedi M, Tabarin A, Roger P (1999). Abdominal obesity increases overnight cortisol excretion. J Endocrinol Invest.

[B20] Korbonits M, Trainer PJ, Nelson ML, Howse I, Kopelman PG, Besser GM, Grossman AB, Svec F (1996). Differential stimulation of cortisol and dehydroepiandrosterone levels by food in obese and normal subjects: relation to body fat distribution. Clin Endocrinol.

[B21] Ljung T, Andersson B, Bengtsson BA, Björntorp P, Mårin P (1996). Inhibition of cortisol secretion by dexamethasone in relation to body fat distribution: a dose-response study. Obes Res.

[B22] Björntorp P, Rosmond R (2000). Neuroendocrine abnormalities in visceral obesity. Int J Obes Relat Metab Disord.

[B23] Travison TG, O'Donnell AB, Araujo AB, Matsumoto AM, McKinlay JB (2007). Cortisol levels and measures of body composition in middle-aged and older men. Clin Endocrinol.

[B24] The National Board of Health and Welfare, Sweden. Statistics on health, medical care utilisation, social conditions, and social service. How is Sweden doing? (in Swedish). http://192.137.163.40/epcfs/index.asp?modul=hms.

[B25] Statistics Sweden. The Swedish population, comparisons between counties. Population Statistics; 2008 (in Swedish). http://www.scb.se/Pages/ProductTables____25795.aspx.

[B26] Nyholm M, Gullberg B, Haglund B, Råstam L, Lindblad U (2008). Higher education and more physical activity limit the development of obesity in a Swedish rural population. The Skaraborg Project. Int J Obes.

[B27] WHO Study Group (1985). Diabetes mellitus. Tech Rep Ser 727.

[B28] Garde AH, Hansen AM (2005). Long-term stability of salivary cortisol. Scand J Clin Lab Invest.

[B29] Otte C, Yassouridis A, Jahn H, Maass P, Stober N, Wiedemann K, Kellner M (2003). Mineralocorticoid receptor-mediated inhibition of the Hypothalamic-Pituitary-Adrenal axis in aged humans. J Gerontol A Biol Sci Med Sci.

[B30] Wilkinson CW, Peskind ER, Raskind MA (1997). Decreased hypothalamic-pituitary-adrenal axis sensitivity to cortisol feedback inhibition in human aging. Neuroendocrinology.

[B31] Pedersen SB, Jonler M, Richelsen B (1994). Characterization of regional and gender differences in glucocorticoid receptors and lipoprotein lipase activity in human adipose tissue. J Clin Endocrinol Metab.

[B32] Andrew R, Phillips DI, Walker BR (1998). Obesity and gender influence cortisol secretion and metabolism in men. J Clin Endocrinol Metab.

[B33] Stewart PM, Boulton A, Kumar S, Clark PM, Shackleton CH (1999). Cortisol metabolism in human obesity: Impaired cortisone→cortisol conversion in subjects with central adiposity. J Clin Endocrinol Metab.

[B34] Vierhapper H, Nowotny P, Waldhausl W (2004). Production rates of cortisol in obesity. Obes Res.

[B35] Björntorp P (1991). Visceral fat accumulation: the missing link between psychosocial factors and cardiovascular disease?. J Intern Med.

[B36] Epel ES, McEwen B, Seeman T, Matthews K, Castellazzo G, Brownell KD, Bell J, Ickovics JR (2000). Stress and body shape: stress-induced cortisol secretion is consistently greater among women with central fat. Psychosom Med.

[B37] Björntorp P, Holm G, Rosmond R (1999). Hypothalamic arousal, insulin resistance and Type 2 diabetes mellitus. Diabet Med.

[B38] Heim C, Ehlert U, Hellhammer DH (2000). The potential role of hypocortisolism in the pathophysiology of stress-related bodily disorders. Psychoneuroendocrinology.

[B39] Clasey JL, Bouchard C, Teates CD, Riblett JE, Thorner MO, Hartman ML, Weltman A (1999). The use of anthropometric and dual-energy X-ray absorptiometry (DXA) measures to estimate total abdominal and abdominal visceral fat in men and women. Obes Res.

[B40] Stanforth PR, Jackson AS, Green JS, Gagnon J, Rankinen T, Desprès JP, Bouchard C, Leon AS, Rao DC, Skinner JS, Wilmore JH (2004). Generalized abdominal visceral fat prediction models for and white adults aged 17–65 y: the HERITAGE Family Study. Int J Obes.

